# Global prevalence of *Borrelia burgdorferi* and *Anaplasma phagocytophilum* coinfection in wild and domesticated animals: A systematic review and meta-analysis

**DOI:** 10.7189/jogh.14.04231

**Published:** 2024-12-06

**Authors:** Weijie Ma, Li Gao, Xinya Wu, Lei Zhong, Xun Huang, Rui Yang, Hanxin Wu, Liangyu Zhu, Weijiang Ma, Li Peng, Bingxue Li, Jieqin Song, Suyi Luo, Fukai Bao, Aihua Liu

**Affiliations:** 1 Yunnan Province Key Laboratory of Children’s Major Diseases Research, Department of Pathogen Biology and Immunology, School of Basic Medical Sciences, Kunming Medical University, China; 2 Yunnan Provincial Key Laboratory of Public Health and Biosafety, School of Public Health, Kunming Medical University, China

## Abstract

**Background:**

Both *Borrelia burgdorferi* (*Bb*) and *Anaplasma phagocytophilum* (*Ap*) can infect humans and animals through tick-borne transmission, resulting in zoonosis. Under certain conditions, human infection can lead to Lyme disease (LD) and human granulocytosis (HGA), whereas infection in animals can cause various acute and non-specific symptoms. The combination of *Bb* and *Ap* has been reported to increase the disease severity in infected animals. In this systematic review and meta-analysis, we investigated the global diversity of *Bb* and *Ap* coinfection in animals and their prevalence and distribution regarding spatial and species ecoepidemiology.

**Methods:**

We queried PubMed, Web of Science, Embase, and the Cochrane Library for original studies on *Bb* and *Ap* coinfection. We assessed the rate of *Bb* and *Ap* in all included articles by single-group meta-analysis and subgroup analyses. We evaluated publication bias using a combination of funnel plots, Egger’s tests, and Begg’s tests, and conducted risk of bias assessment using the SYRCLE tool.

**Results:**

Our search retrieved 40 articles, with eight involving 8419 infected animals meeting our inclusion criteria. The SYRCLE bias risk assessment indicated that most of the included studies were of high quality. Forest maps showed that the combined *Bb* and *Ap* infection rate in animals worldwide was 5.5% (95% confidence interval (CI) = 2.4–9.6). Subgroup analysis of forest maps showed that the coinfection rates were 8.2% (95% CI = 2.2–17.2) in North American, 0.2% (95% CI = 0.1–0.7) in European, and 1.2% (95% CI = 0.8–1.8) in Asian animals. Coinfection rates were 6.7% (95% CI = 2.7–12.2) in domestic and 0.0% (95% CI = 0.0–0.4) in wild animals. The coinfection rates were 9% (95% CI = 5.7–12.8) in domestic horses and 6% (95% CI = 1.9–12.2) in domestic dogs, whereas 7.5% (95% CI = 3–17.9) in wild squirrels and 0.2% (95% CI = 0.1–0.7) in wild mice. Funnel diagrams, Egger’s tests, and Begg’s tests showed no significant publication bias in the included studies.

**Conclusions:**

Spatial epidemiology showed that coinfection with *Bb* and *Ap* in animals worldwide was most prevalent in the southwestern region of North America, whereas species epidemiology showed that coinfection was most prevalent in domesticated horses, followed by wild squirrels and domesticated dogs, but was less common in wild mice. These data on the epidemiological status of *Bb* and *Ap* coinfection in animals may help guide the prevention and treatment of zoonosis.

*Borrelia burgdorferi* (*Bb*) is a tick-borne obligate parasitic pathogen whose natural hosts include cattle, horses, dogs, cats, deer, raccoons, wolves, hares, foxes, and a variety of small rodents [1–4]. These animals often present with acute symptoms, including fever, lameness, and joint swelling, as well as with chronic symptoms, such as behavioural changes, dysphagia, and encephalitis [5]. Tick-borne infection of humans with *Bb* can cause Lyme disease (LD), which is characterised by erythema migrans, involvement of the nervous system or heart, and arthritis [6].

*Anaplasma phagocytophilum* (*Ap*) is an obligate gram-negative intracellular bacterium with a wide range of hosts, including many species of domestic and wild animals, with various symptoms, such as high fever, anorexia, lethargy, hyperesthesia, and lameness [7,8]. *Ap* infection in humans can cause an acute febrile illness called human granulocytic anaplasmosis (HGA). Clinical manifestations range from asymptomatic infection to fatal disease, depending on age and coinfection, with main non-specific symptoms including fever, chills, malaise, headache, and myalgia [9,10].

Ticks are strict blood-sucking arthropods of the order *Ixoidea* that can carry various infectious agents, including *Bb* and *Ap* [11]. Following host co-feeding or ingestion, ticks can be transmitted by the blood throughout host bodies [11]. Two or more tick-borne pathogens can invade a host at the same time or successively, resulting in coinfection and the development of tick-borne infectious diseases [12]. Although *Bb* and *Ap* have been detected separately in several vertebrate species, coinfection with them has not been systematically or comprehensively analysed [13,14]. Coinfection of humans or other vertebrate species with *Bb* and *Ap* may increase the severity of the disease and have serious consequences in its diagnosis and treatment [15]. Determining the prevalence of *Bb* and *Ap* coinfection in different regions and different species may help in understanding the epidemic characteristics of tick-borne infectious diseases and provide a scientific basis for disease prevention.

In this systematic review and meta-analysis, we aim to assess the prevalence of *Bb* and *Ap* coinfection and the characteristics of its prevalence across regions, countries, and species. As *Bb* and *Ap* can cause zoonotic diseases in and affect the health of humans and animals, as well as lead to economic losses, our study could fill the gap in this part of the research and provide a better direction and basis for the prevention and treatment of zoonotic diseases.

## METHODS

In this study, we followed the PRISMA guidelines [16] and registered the study with PROSPERO (CRD42024488954).

### Search strategy

We systematically searched PubMed, Web of Science, Embase, and Cochrane Library for original articles on animals published in English until 3 November 2023. We designed the strategy using a combination of terms such as ‘*Borrelia burgdorferi*’, ‘*Anaplasma phagocytophilum*’, ‘coinfection’, ‘animals’, and ‘prevalence’ and their related keywords (Table S1 in the **Online Supplementary Document**).

### Inclusion and exclusion criteria

We imported all retrieved articles into EndNote, version 20. We included studies that described wild or domesticated animals simultaneously infected with *Bb* and *Ap* and excluded them if they were case reports, studies in humans, non-original studies (letters, reviews, editorials), or if they did not clearly describe the total number of animals and the number coinfected.

### Data screening and extraction

Two researchers (MW and GL) independently selected articles, extracted data, and evaluated article quality, with any disagreements resolved through discussion. After removing duplicate articles, we reviewed the titles and abstracts of the remaining articles and selected eligible articles based on the inclusion and exclusion criteria. We downloaded and read the full text of each of the selected articles. We extracted data from each of these articles into Microsoft Excel tables, which included the name of the first author, year of publication, article title, country of origin, species of animals coinfected with *Bb* and *Ap*, numbers of animals infected individually with *Bb* and *Ap*, and numbers of animals coinfected with *Bb* and *Ap*.

### Assessment of bias risk

We assessed the quality of the included studies using the SYRCLE risk of bias tool, which consists of six components (i.e. selection bias, performance bias, detection bias, loss of follow-up bias, reporting bias, and other biases) and 10 items [17]. We generated bias risk maps and charts using R, version 4.3.2 (R Core Team, Vienna, Austria).

### Statistical analysis

We used the ‘metaprop’ package in STATA, version 17.0 (StataCorp LLC, College Station, Texas, USA) to determine the rate of *Bb* and *Ap* coinfection and calculate standard errors by dividing the number of coinfected animals by the total number of animals in the included studies, and to determine coinfection rates in different regions of the world and different species. ‘Metaprop’ is a package without invalid lines, specially developed for the meta-analysis of single-group rates, which is characterised by rates not being affected by data characteristics when the ‘metaprop’ function is used to merge rates [18,19]. Using the single-group rate meta-analysis method [20], we calculated 95% confidence intervals (CIs) and used the general inverse variance method to establish a random effects model; determined the combined estimates to generate forest plots; and performed heterogeneity tests (e.g. *P*-values of *I^2^* and Cochran’s Q tests) [21,22]. We used a random effects model because of its ability to deal with inter-study heterogeneity, provide more robust and conservative conclusions, and quantify such heterogeneity through statistical tests and heterogeneity assessment.

We also tested the included studies for publication bias. Specifically, we used the ‘meta funnel’ package of STATA software to construct funnel plots, with the effect size odds ratio being the horizontal coordinate and the inverse of the effect size to the numerical standard error 1/standard error (log odds ratio) being the vertical coordinate, and CIs set at 95%. Then, we performed Egger’s and Begg’s tests using the ‘meta bias’ package, with the estimated effect value and its accuracy subjected to weighted regression analysis, and the symmetry of the funnel plot further quantified and evaluated to determine *P*-values [23]. A *P*-value <0.05 was indicative of publication bias, whereas a *P*-value >0.05 was indicative of a lack of publication bias [24].

## RESULTS

### Search results and included studies

**Figure 1:**
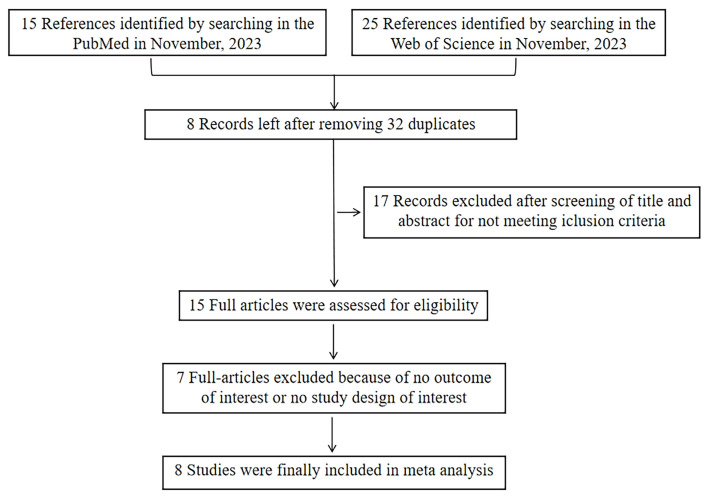
PRISMA flow diagram.

We identified a total of 40 studies through PubMed (n = 15), Web of Science (n = 25), Embase (n = 0), and Cochrane Library (n = 0) (**Figure 1**). We screened their titles and abstracts, after which we excluded eight duplicates and 17 studies which we deemed ineligible. We then read the full text of the 15 remaining studies; of these, we excluded seven that did not meet the inclusion and exclusion criteria. Eight studies met our criteria and were included in the meta-analysis.

### Characteristics and quality assessment of included studies

Of the eight included studies, six were conducted in the USA, one in Europe (Hungary), and one in South Korea (**Table 1**). They were published between 2010 and 2023 and included 8419 animals, of which 206 (2.4%) were coinfected with *Bb* and *Ap*. Based on the descriptions in the included articles and our estimates of whether the animals included in the study had been domesticated and controlled by humans, as well as their relationship and interaction with humans, we divided them into wild animals and domestic animals. Of the latter, 180 were domestic animals and 24 were wild animals, with these animals including 23 horses, 176 dogs, four squirrels, and three mice.

**Table 1:** Characteristics of included studies

Study ID	Species of animals	Country	Total number of animals	*Bb* and *Ap* coinfection in all animals
Nieto et al., 2010 [25]	Squirrels	California	53	4
Johnson et al., 2011 [26]	Dogs	Hungary	150	22
Farkas et al., 2014 [27]	Mice	Camp Ripley, Minnesota	1305	3
Laamari et al., 2020 [28]	Horses	America	128	10
Laamari et al., 2020 [28]	Horses	America	128	13
Lee et al., 2020 [29]	Dogs	Korea	2215	27
Mahachi et al., 2020 [30]	Dogs	America	214	66
Meyers et al., 2021 [31]	Dogs	Northeast America	476	1
Hazelrig et al., 2023 [32]	Dogs	Eastern America	3750	60

*Ap* – *Anaplasma phagocytophilum*, *Bb* – *Borrelia burgdorferi*

Of the eight included articles, one used two methods to detect coinfection with *Bb* and *Ap*, with all eight being prospective studies. Based on our risk of bias assessment, we found that three studies were ‘low risk’, four were ‘medium risk’, and one was ‘high risk’ (**Table 2**; Figure S1 in the **Online Supplementary Document**). Therefore, most of these studies were of high quality, indicating that our findings are reliable.

**Table 2:** Risk of bias of the included studies

Study ID	Selection bias	Performance bias	Detection bias	Loss of follow-up bias	Reporting bias	Other bias	Overall
Nieto et al., 2010 [25]	Low	Unclear	Unclear	Low	Unclear	Low	Unclear
Johnson et al., 2011 [26]	Low	Low	Low	Low	Unclear	Low	Low
Farkas et al., 2014 [27]	Low	Unclear	Unclear	Low	Unclear	Low	Unclear
Laamari et al., 2020 [28]	Low	Unclear	Unclear	Low	Unclear	Low	Unclear
Lee et al., 2020 [29]	Low	Unclear	Unclear	Low	Unclear	High	High
Mahachi et al., 2020 [30]	Low	Low	Low	Low	Unclear	Low	Low
Meyers et al., 2021 [31]	Low	Unclear	Unclear	Low	Unclear	Low	Unclear
Hazelrig et al., 2023 [32]	Low	Unclear	Low	Low	Unclear	Low	Low

### Meta-analysis of *Bb* and *Ap* coinfection in animals

**Figure 2:**
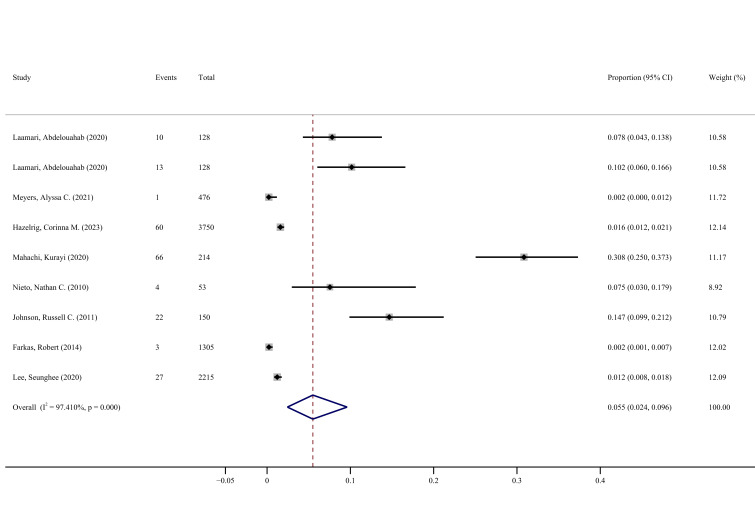
Forest plots of *Bb* and *Ap* coinfection rates in all included studies. *Ap* – *Anaplasma phagocytophilum*, *Bb* – *Borrelia burgdorferi*, CI – confidence interval.

**Figure 3:**
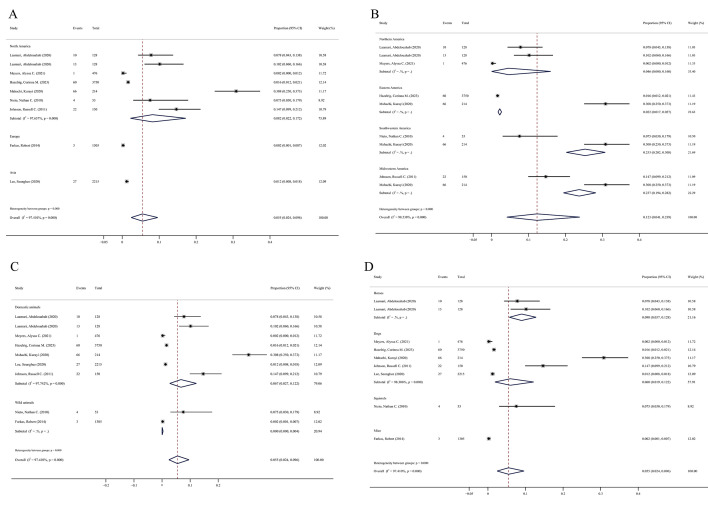
Forest maps of *Bb* and *Ap* coinfection in different geographic regions and animals. **Panel A.** North America, Europe, and Asia. **Panel B.** Different regions of the US. **Panel C.** Domestic and wild animals. **Panel D.** Different species.

Forest maps of all included studies showed that the rate of *Bb* and *Ap* coinfection in all the animals in the included studies, calculated according to random-effects models, was 5.5% (95% CI = 2.4–9.6) (**Figure 2**). The prevalence and characteristics of *Bb* and *Ap* coinfection in different regions and different species were determined by subgroup analyses, enabling a prediction of possible causes of coinfection with these two pathogens (**Figure 3**, **Table 3**).

**Table 3:** Subgroup analysis of *Bb* and *Ap* coinfection in different regions and animals

	Coinfection rate, % (95% CI)	Weight, %
Continents		
*North America*	8.20 (2.20–17.20)	75.89
*Europe*	0.20 (0.10–0.70)	12.02
*Asia*	1.20 (0.80–1.80)	12.09
American areas		
*Northern America*	4.60 (0.00–16.00)	33.40
*Eastern*	2.20 (1.70–2.70)	22.63
*Southwestern*	25.3 (20.20–30.80)	21.69
*Midwestern*	23.7 (19.40–28.20)	22.29
Animals		
*Domestic animals*	6.70 (2.70–12.20)	79.06
*Wild animals*	0.00 (0.00–0.40)	20.94
Species		
*Horses*	9.00 (5.70–12.80)	21.16
*Dogs*	6.00 (1.90–12.20)	57.91
*Squirrels*	7.50 (3.00–17.90)	8.92
*Mice*	0.20 (0.10–0.70)	12.02

*Ap* – *Anaplasma phagocytophilum*, *Bb* – *Borrelia burgdorferi*, CI – confidence interval

#### Meta-analysis of *Bb* and *Ap* coinfection by geographic regions

The rates of *Bb* and *Ap* coinfection in articles from North America (US) were 8.2% (95% CI = 2.2–17.2), 0.2% (95% CI = 0.1–0.7) from Europe (Hungary), and 1.2% (95% CI = 0.8–1.8) from Asia (South Korea) (**Figure 3**, Panel A and **Table 3**). These results indicated that *Bb* and *Ap* coinfection in animals is highly prevalent in North America but is less prevalent in Asia and Europe.

The six studies from North America included 5327 animals: 732 in the northern, 3964 in the eastern, 267 in the southwestern, and 364 in the midwestern USA. The rates of *Bb* and *Ap* coinfection in northern USA populations were 4.6% (95% CI = 0–16), 2.2% (95% CI = 1.7–2.7) in eastern, 25.3% (95% CI = 20.2–30.8) in southwestern, and 23.7% (95% CI = 19.4–28.2) in midwestern US populations (**Figure 3**, Panel B and **Table 3**). These findings indicated that *Bb* and *Ap* coinfection animals are most highly prevalent in the southwestern USA, followed by the midwestern, northern, and eastern USA.

This meta-analysis showed that *Bb* and *Ap* coinfection was more prevalent in North America, especially in the southwestern region of the USA than in other regions of the world.

#### Meta-analysis of *Bb* and *Ap* coinfection by species

The eight studies included 7061 domestic animals (horses and dogs) and 1358 wild animals (squirrels and mice). The rates of *Bb* and *Ap* coinfection in domestic animals were 6.7% (95% CI = 2.7–12.2) and 0.0% (95% CI = 0.0–0.4) in wild animals, indicating that *Bb* and *Ap* coinfection is more prevalent in domestic than in wild animals (**Figure 3**, Panel C and **Table 3**,).

The 7061 domestic animals included 256 horses and 6805 dogs in domestic animals, with 23 horses (9%; 95% CI = 5.7–12.8) and 176 dogs (6%; 95% CI = 1.9–12.2) being positive for *Bb* and *Ap* coinfection. Of the 1358 wild animals, four squirrels (7.5%; 95% CI = 3–17.9) and three mice (0.2%; 95% CI = 0.1–0.7) were positive for *Bb* and *Ap* coinfection (**Figure 3**, Panel D and **Table 3**,).

Taken together, these results indicate that, to improve the accuracy of the effect estimate coinfection is more prevalent in domestic than in wild animals globally. Coinfection was especially prevalent in horses, followed by wild squirrels, dogs, and mice.

### Publication bias

**Figure 4:**
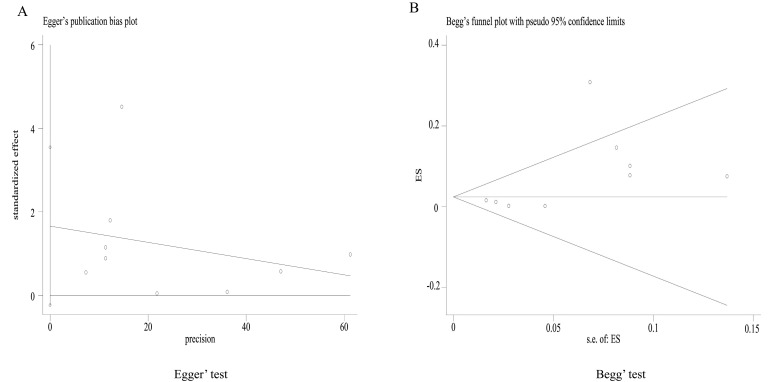
Egger’s test and Begg’s test funnels of all included studies. **Panel A.** Egger’s test funnel of all included studies. **Panel B.** Begg’s test funnel of all included studies. ES – logarithm of the odds ratio, s.e. – standard error

A funnel plot showed that the included studies were evenly distributed on both sides of the combined effect size, as shown by the line perpendicular to the x-axis, and were symmetrically distributed across the 95% CI (Figure S2 in the **Online Supplementary Document**). One study, however, fell outside the 95% CI, indicating some heterogeneity in this meta-analysis. Meanwhile, both Egger’s test and Begg’s test showed no significant publication bias (*t* = 2.07; *P* = 0.077) (**Figure 4**, Panels A and B). This would suggest that the results of this meta-analysis were robust.

## DISCUSSION

**Figure 5:**
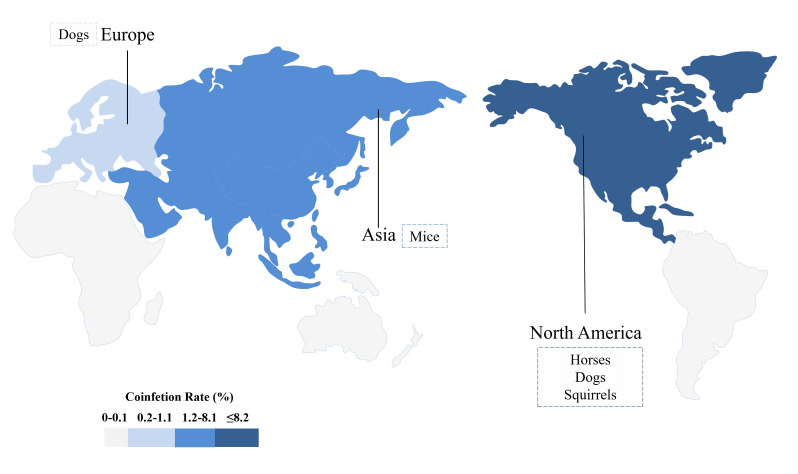
*Bb* and *Ap* coinfection of different species in different continents. Different colours represent the number of animals coinfected with *Bb* and *Ap* on different continents. *Ap* – *Anaplasma phagocytophilum*, *Bb* – *Borrelia burgdorferi*.

To our knowledge, this is the first study to explore the prevalence of *Bb* and *Ap* coinfection in wild and domestic animals worldwide. Based on published evidence on prevalence, incidence, and risk factors, our findings outline the global epidemiology of the prevalence and distribution of risk factors associated with *Bb* and *Ap* coinfection across species and geographic locations. Coinfection rates of *Bb* and *Ap* seem to be highest in the southwestern region of the US, higher in domestic animals than in wild animals, and most prevalent in domesticated horses, followed by squirrels, dogs, and rats (**Figure 5**).

Understanding the geographic and species distribution of *Bb* and *Ap* coinfection is necessary for the prevention and control of zoonosis [33,34]. Since *Bb* and *Ap* are transmitted by the same vector, HGA frequently occurs in regions where LD is endemic, particularly in North America and Europe, where it is most prevalent [35]. Moreover, an increasing number of tick-exposed patients are being identified as coinfected [36]. We further found that *Bb* and *Ap* coinfected animals are highest in the southwestern region of the USA, giving us reason to suspect that the distribution of *Bb* and *Ap* coinfected humans is also changing, which provides a new direction for the prevention and control of zoonotic diseases. In addition, because *Bb* shares a common carrier with *Ap*, ticks coinfected with *Bb* and *Ap* have inevitably been found in these areas, and as with other tick-borne infectious diseases, HGA and LD can prevent infection by reducing exposure to ticks [37].

Several studies evaluating animals separately infected with these pathogens found that the prevalence of each was significantly higher in wild than in domestic animals, possibly due to their closer proximity to tick vectors and the lack of control measures in their natural habitats [38,39]. In contrast, we found that the coinfection rate of *Bb* and *Ap* was significantly higher in domestic than in wild animals and that coinfection was most prevalent in domesticated horses. This suggests that both domestic and wild animals may play important roles in the epidemiology of *Bb* and *Ap* coinfection, but that domestic animals may be more important pathogen hosts. Animals are more likely to encounter ticks than humans because they tend to spend more time outdoors, are closer to the ground and vegetation, and have a coat that is easily attached to ticks [40]. Because domestic animals live in habitats closer to that of humans than wild animals and share the same living spaces and outdoor areas as humans, measures must be taken to reduce the rate of human and animal infection.

Although natural simultaneous exposure to *Bb* and *Ap* has been detected in animals such as sheep and dogs, it complicates clinical presentation, diagnosis, and therapeutic response in animals, leading to more severe blood abnormalities and enhancing the pathogenic process [41]. In humans, coinfection with *Bb* and *Ap* can lead to more severe complications and longer duration of clinical symptoms [42]. It is unclear, however, whether several clinical signs and clinicopathological abnormalities are associated with coinfection, indicating the need for studies evaluating possible background mechanisms [43,44]. Our results indicate a significant risk of co-transmission of *Bb* and *Ap* pathogens through tick bites, emphasising the need for serious attention. These findings suggest that prevention and control strategies for zoonotic diseases should be tailored to specific regions and species. However, further investigation into other potential ecological and environmental factors is warranted.

One of the strengths of this study is our search of four large databases, with the included articles covering three continents: North America, Asia, and Europe. Another is the use of SYRCLE, the first tool to be adjusted from Cochrane’s Risk of Bias Tool to specifically target animal experiments [18]. Our assessment showed that the included studies had good quality, adding to the robustness of our findings. We also evaluated the studies for publication bias using funnel plots, Egger’s test, and Begg’s test, ensuring the comprehensiveness of our evaluation.

This study, however, also has several limitations. First, the forest plots showed substantial heterogeneity, which affected the interpretation of the results. Nevertheless, we performed subgroup analyses to identify potential sources of heterogeneity. Neither the overall assessment of all included studies nor the subgroup analysis identified specific sources of heterogeneity. However, this analysis may have been influenced by several factors. First, we only included studies published in English, so we may have overlooked important studies published in other languages, potentially leading to publication bias. Additionally, the lack of available research data might have also contributed to the high heterogeneity observed in the study. Few studies to date have evaluated coinfection in *Bb* and *Ap* animals. Some articles excluded from this study did not report sample sizes for coinfections, reducing the available data. Second, we found no studies from regions such as South America, Africa, and Oceania. Prevalence data were available only for North America, Asia, and Europe, with the included studies primarily focussing on North America, particularly the USA. This focus on specific geographic areas and animal species limits the generalisability and applicability of our findings. The prevalence and characteristics of *Bb* and *Ap* coinfection in these areas without study data deserve further exploration in the future. Third, we explored geographic differences in infection rates, but did not further analyse potential ecological or environmental factors for these differences, such as climate, tick density, and wildlife interaction patterns. Future research on these factors is highly recommended, as it could offer a more comprehensive understanding of this topic. Given these limitations, our results should be interpreted with caution.

## CONCLUSIONS

To our knowledge, this is the first and most comprehensive systematic review and meta-analysis of the global prevalence of Bb and Ap coinfection in wild and domesticated animals. The rate of *Bb* and *Ap* coinfection in animals were the highest in the southwestern region of the USA, while coinfection was generally more common in domestic than in wild animals, with the highest prevalence in domesticated horses, followed by squirrels, dogs, and mice. Although these results may improve our understanding of the epidemiology of *Bb* and *Ap* coinfection and provide a better basis for the prevention and treatment of zoonosis, given the small number of studies and regional limitations, we urge readers to adopt a more cautious interpretation of our findings.

## Additional material


Online Supplementary Document
