# Supplementary material for: Global prevalence of Borrelia burgdorferi and Anaplasma phagocytophilum coinfection in wild and domesticated animals: A systematic review and meta-analysis
Source: J Glob Health. 2024 Dec 6;14:04231. doi: 10.7189/jogh.14.04231 (PMC11622344; doi:10.7189/jogh.14.04231)

Table S1. The search strategy in each database and results.

Database	Search strategy	Date	Result
PubMed	(((((("Borrelia burgdorferi") OR ("Lyme Disease Spirochete")) OR ("Borrelia burgdorferi sensu stricto")) AND (Anaplasma)) AND ((("co infction") OR (co infection)) OR (coinfection))) AND ((animals) OR (animal))) AND ((((((((prevalence) OR (Prevalences)) OR ("Period Prevalence")) OR ("Period Prevalences")) OR ("Prevalence, Period")) OR ("Point Prevalence")) OR ("Point Prevalences")) OR ("Prevalence, Point"))	2023/11/3	15
Web of Science	1 ((TS=("Borrelia burgdorferi")) OR TS=("Lyme Disease Spirochete")) OR TS=("Borrelia burgdorferi sensu stricto")<br>2 TS=(Anaplasma)<br>3 ((TS=(co-infection)) OR TS=("co infction")) OR TS=(coinfection)<br>4 (TS=(animals)) OR TS=(animal)<br>5 (((((((TS=(prevalence)) OR TS=(Prevalences)) OR TS=("Period Prevalence")) OR TS=("Period Prevalences")) OR TS=("Prevalence, Period")) OR TS=("Point Prevalence")) OR TS=("Point Prevalences")) OR TS=("Prevalence, Point")<br>1 and 2 and 3 and 4 and 5	2023/11/3	25

Continued

Database	Search strategy	Date	Result
Embase	1 'borrelia burgdorferi':ab,ti OR 'lyme disease spirochete':ab,ti OR 'borrelia burgdorferi sensu stricto':ab,ti<br>2 anaplasma:ab,ti<br>3 prevalence:ab,ti OR prevalences:ab,ti OR 'period prevalence':ab,ti OR 'period prevalences':ab,ti OR 'prevalence, period':ab,ti OR 'point prevalence':ab,ti OR 'point prevalences':ab,ti OR 'prevalence, point':ab,ti<br>4 animal:ab,ti OR animals:ab,ti<br>5 coinfction:ab,ti OR 'co infction':ab,ti<br>1 and 2 and 3 and 4 and 5	2023/11/3	0
Cochrane Library	1 (Borrelia burgdorferi):ti,ab,kw OR (Lyme Disease Spirochete):ti,ab,kw OR (Borrelia burgdorferi sensu stricto):ti,ab,kw<br>2 MeSH descriptor: [Anaplasma] explode all trees<br>3 (co infction) OR (co-infction) OR (coinfction)<br>4 (animals):ti,ab,kw OR (animal):ti,ab,kw<br>5 (prevalence):ti,ab,kw OR (Prevalences):ti,ab,kw OR (Period Prevalence):ti,ab,kw OR (Period Prevalences):ti,ab,<br>kw OR (Prevalence, Period):ti,ab,kw OR (Point Prevalence):ti,ab,kw OR (Point Prevalences):ti,ab,kw OR (Prevalence, Point):ti,ab,kw<br>1 and 2 and 3 and 4 and 5	2023/11/3	0

Figure S1. Risk of bias presented as percentages across included studies.

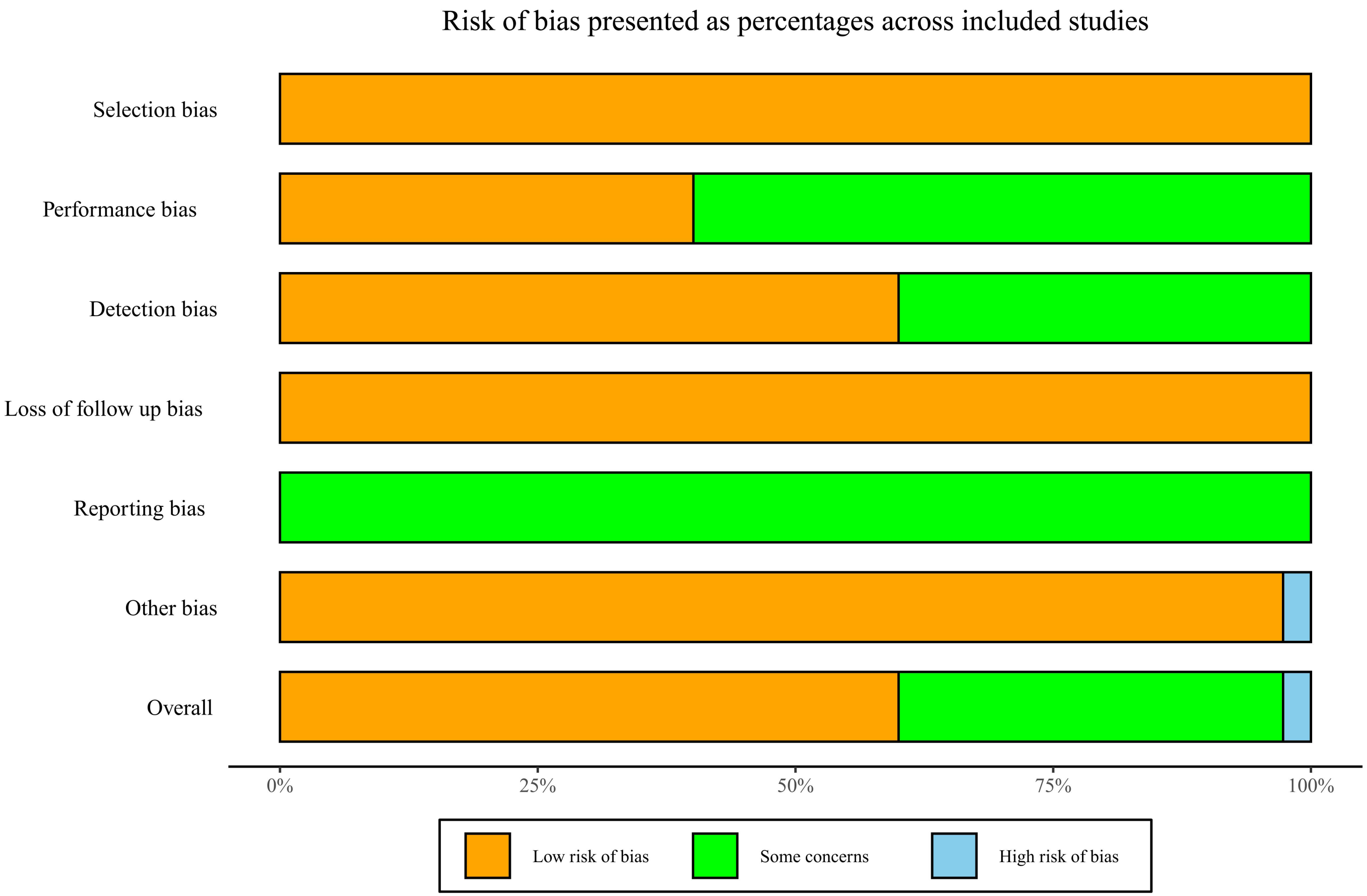

Figure S2. Funnel plot of all included studies.

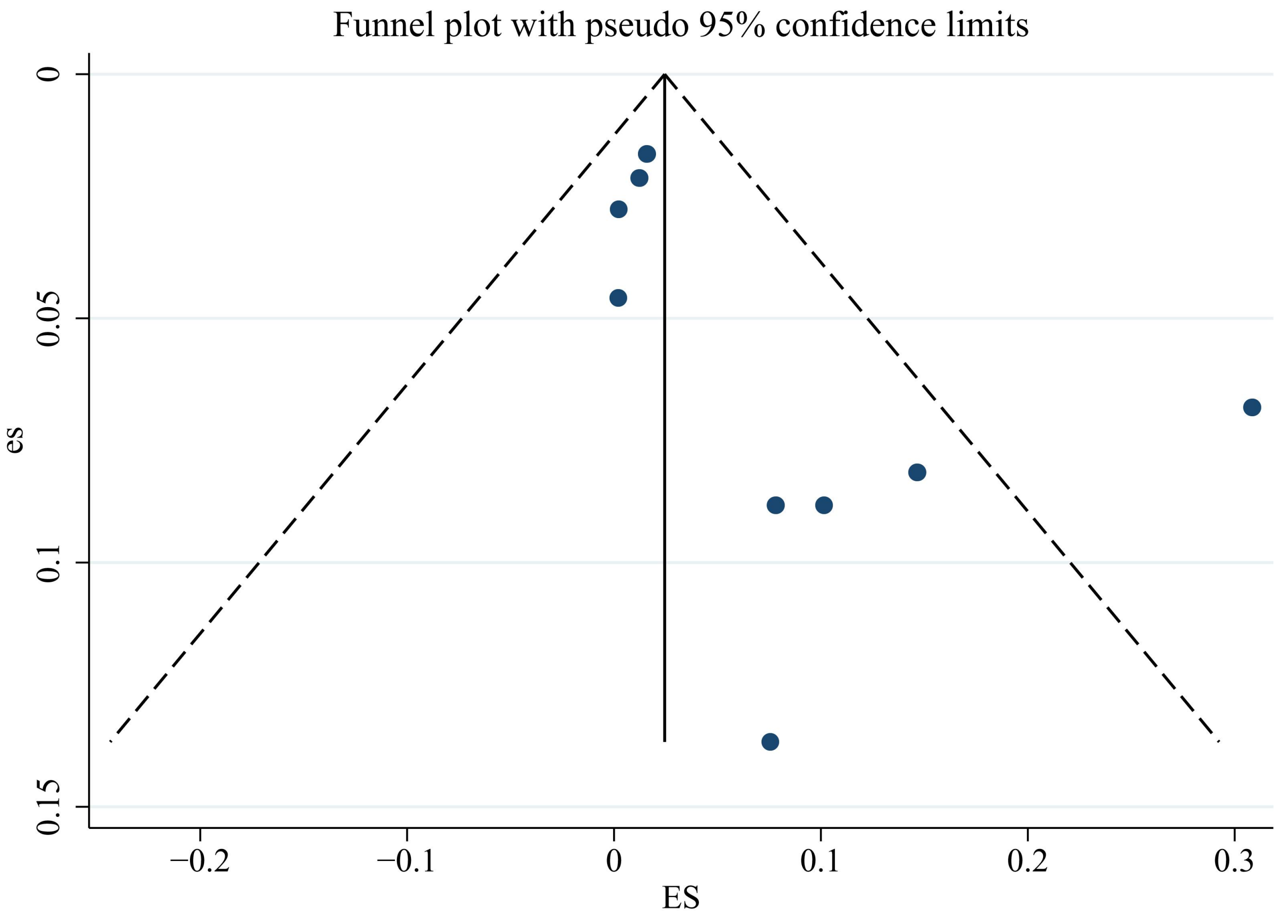

Supplement: Online Supplementary Document [file jogh-14-04231-s001.pdf]
